# Treatment intensity affects immune reconstitution even after childhood cancer not treated with hematopoietic stem cell transplantation

**DOI:** 10.1002/cnr2.2069

**Published:** 2024-05-20

**Authors:** Ella Antikainen, Marika Grönroos, Anu Huurre, Laura Korhonen, Ville Peltola, Päivi Lähteenmäki, Linnea Schuez‐Havupalo

**Affiliations:** ^1^ Institute of Dentistry University of Turku Turku Finland; ^2^ Department of Pediatrics and Adolescent Medicine Turku University Hospital and University of Turku Turku Finland

**Keywords:** chemotherapy, children, immune reconstitution, malignant diseases

## Abstract

**Background:**

Only a few previous studies examine immune system recovery after completed cancer treatment.

**Aims:**

The aim of this study was to analyze immune reconstitution after childhood cancer therapy in a non‐hematopoietic stem cell transplantation setting.

**Methods and results:**

We analyzed children (*N* = 79) who received chemotherapy with/without irradiation for cancer diagnosed between 2014 and 2019 at Turku University Hospital, Finland. We retrospectively collected data on baseline parameters and post‐treatment immunological recovery, namely neutrophil and lymphocyte counts, IgG levels, CD19, CD4 and natural killer cell counts. Immunological parameters were followed until their normalization. Treatment intensity was stratified according to the Intensity of Treatment Rating Scale (ITR‐3). We analyzed the effects of treatment intensity on normalization of immunological parameters across the entire treatment range. Treatment intensity had a major effect on immune system recovery after completion of treatment. Most patients had normal immunological parameters 1–4 months post‐treatment both in high‐ and low‐intensity treatment groups, but patients classified in the high‐intensity group had low parameters more often than patients in the low‐intensity group.

**Conclusion:**

Our data suggest a fast recovery of studied immunological parameters after the majority of current pediatric oncologic treatments. Treatment for high‐risk acute lymphoblastic leukemia, acute myeloid leukemia, medulloblastoma, and mature B‐cell lymphoma was associated with prolonged recovery times for a substantial proportion of cases. High treatment intensity was associated with prolonged immunological recovery.

## 1 INTRODUCTION

Pediatric cancer treatments have greatly advanced during the last decades. However, these treatments are associated with numerous toxicities including effects on the immune system. The vast majority of studies examining reconstitution of the immune system after childhood cancer are carried out in patients after hematopoietic stem cell transplantation (HSCT). There are some studies following patients after conventional chemotherapy.^1^, ^2^, ^3^, ^4^, ^5^, ^6^, ^7^, ^8^, ^9^, ^10^, ^11^, ^12^, ^13^, ^14^, ^15^, ^16^, ^17^

Most of the studies having been performed in a non‐transplant setting examine immune reconstitution after therapy in patients with acute lymphoblastic leukemia (ALL).^2^, ^4^, ^5^, ^8^, ^9^, ^10^, ^11^, ^13^, ^15^, ^16^, ^17^ While there is an existing body of research in this field, findings are not always consistent. ALL treatments have developed over time, which means that continuous monitoring of post‐treatment immunological effects is required. Studies on immune system recovery after treatment for solid tumors in patients not having received high‐dose therapy with a stem cell transplant are rare.^1^, ^6^, ^9^, ^12^, ^14^ Findings from patients with ALL cannot be directly transferred to other populations, since ALL has distinct features with regard to immunological recovery. The disease itself affects precursors of the B‐ or T cell lines, duration of treatment is long in comparison to treatment of solid tumors, and treatment modalities, such as corticosteroid courses, cause pronounced lymphodepletion and impairment of thymus function.

Immunosuppressed patients may experience serious, or even lethal infections as a complication of cancer treatment. While profound neutropenia with its associated risks of developing sepsis is an important concern in the acute setting of cancer treatments, more subtle deviations of innate or adaptive immune functions may be associated with infection risks to the pediatric patient even after completion of cancer therapy. Immunosuppressed malignancy patients are often treated under isolation precautions in order to prevent transmission of infections. Depending on the situation, some form of isolation may encompass out‐of‐hospital life to prevent social activities, use of public transport, day‐care, or even school‐attendance. Isolation policies thereby affect the everyday life of the patient's entire family and may result in an isolated lifestyle throughout cancer treatment and even thereafter causing a substantial psychological burden to the entire family.

In this study, we aimed to analyze trends of immune reconstitution after a wide range of pediatric cancer treatments, in order to facilitate predictions of necessary isolation precautions for different patient groups post‐treatment. Since treatments varied greatly in intensity, we used a previously validated tool, the Intensity of Treatment Rating Scale (ITR‐3), to stratify between treatment groups.^18^

## 2 PATIENTS AND METHODS

### 2.1 Patients

**FIGURE 1:**
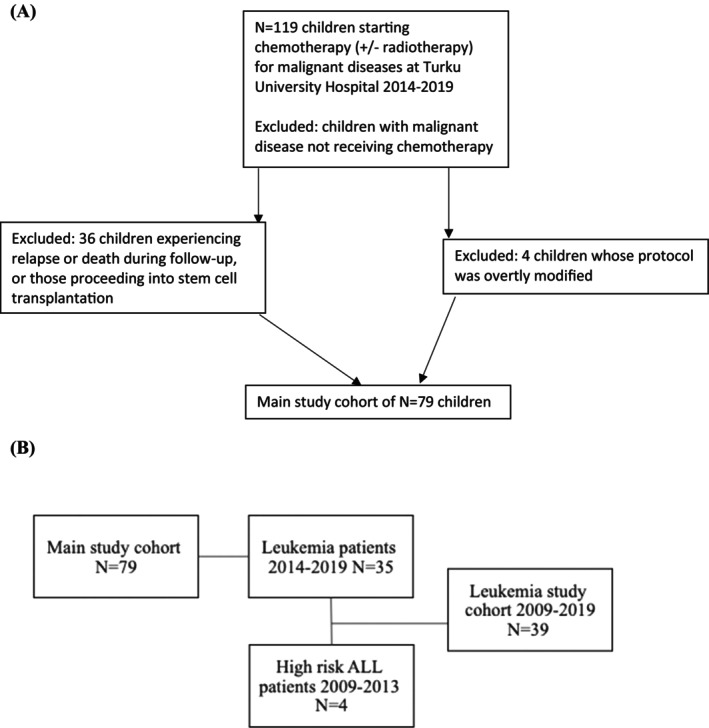
(A) Recruitment to study. Recruitment to the study showing exclusion criteria. (B) Study cohort. Main study cohort: all pediatric patients meeting the study's inclusion criteria who started cytostatic treatment for malignant diseases at Turku University Hospital 2014–2019. The leukemia study cohort consisted of pediatric leukemia patients starting treatment 2014–2019 and high‐risk ALL patients who started treatment 2009–2013.

During the time frame of 2014–2019, there were 119 children starting chemo‐ and/ or radiotherapy treatment for malignant diseases at Turku University Hospital and completing their treatment before 4/2022. There were 79 children who met the following criteria: Children included in this study were treated either by chemotherapy alone (66 patients) or in combination with radiotherapy (13 patients), so chemotherapy was a required treatment modality. Children undergoing stem cell transplantation, children experiencing relapse or death during our follow‐up time, and those whose treatment protocol had been overtly modified were excluded (Figure 1A). All patients were between the ages of 0–16 years at diagnosis.

In a separate dataset (leukemia study cohort), we merged all patients with leukemia diagnosed between 2014 and 2019 with patients with chemotherapy‐treated high‐risk (HR) ALL diagnosed in 2009–2019 (*N* = 39 patients) (Figure 1B).

Cancer treatments were split into two groups based on their intensity. Treatment intensity was stratified according to the ITR‐3.^18^ ITR‐3 categorizes patients into four treatment intensity levels. Given the inclusion criteria to our study, we combined ITR‐3 level 1 + 2 to form the low‐intensity treatment group and ITR‐3 level 3 + 4 to form the high‐intensity treatment group.

### 2.2 Data collection and definitions

Routine blood tests including immunological parameters were taken from all children at around 1‐ and 4‐month post‐treatment and up until their normalization thereafter. Not all subjects had immunological parameters available for both time points (1‐ and 4‐month post treatment), but all had data available at either point depending on the normalization of parameters. Due to these missing data, we merged time points and analyzed whether counts had normalized by 4 months post‐treatment. We furthermore collected data on clinical baseline parameters and treatment‐related aspects. Immunological parameters investigated included plasma immunoglobulin (Ig) G levels and blood neutrophil, lymphocyte, B‐cell (CD19), CD4 T‐cell and natural killer (NK) cell counts.

The IgG in plasma was quantified by immunoturbidimetry (Cobas 8000, Roche Diagnostics). Neutrophil and lymphocyte counts in blood were measured using an automated blood cell counter (Sysmex XE‐2100, Sysmex XN‐10 or XN‐20, Sysmex Inc, Kobe, Japan). The absolute numbers of B cells (CD19+), helper/inducer T cells (CD3+CD4+) and NK cells (CD3−CD16 and CD56+) in peripheral blood were determined by flow cytometry using BD Multitest™ CD3 FITC (clone SK7)/CD8 PE (clone SK1)/CD45 PerCP (clone 2D1)/CD4 APC (clone SK3) and BD Multitest™ CD3 FITC (clone SK7)/CD16 PE (clone B73.1) + CD56 PE (clone NCAM 16.2)/CD45 PerCP (clone 2D1) /CD19 APC (clone SJ25C1) reagents and BD Trucount Tubes™ according to manufacturer's instructions (Becton Dickinson, San Jose, CA, USA). The stained erythrocyte lysed blood samples were acquired and analyzed on BD FACSCanto II flow cytometry using BD FACSCanto™ clinical software or BD FACSVia flow cytometry using BD FACSVia™ clinical software (Becton Dickinson, San Jose, CA, USA). The reference values of plasma IgG, blood neutrophil and lymphocyte counts are based on the assays of the hospital laboratory.

Normalization of results was based on the age‐appropriate reference values for IgG levels. CD19, CD4 and NK cell stratification was based on their normal levels (Tables 2 and 3).

Analyses of CD19 cells and IgG levels excluded patients with rituximab treatment.

### 2.3 Statistics

In our analyses, *p* values less than .05 were regarded as statistically significant.

Background variables were compared between treatment intensity groups using the chi‐square test (Fisher's exact test for small numbers) and Mann–Whitney *U* test. The latter was applied since patients' ages did not follow a normal distribution.

For the leukemia cohort, recovery of immunological parameters was analyzed between treatment groups using the Fisher's exact test. For the main study cohort, the effect of treatment intensity on recovery of immunological outcome measures was first estimated using the Chi‐square test (Fisher's exact test for small numbers). In a second step, adjusted binary logistic regression analysis was performed including all applicable background variables (age, sex, treatment intensity, pre‐existing chronic disease, and previous multiple viral respiratory infections). Apart from treatment intensity, none of the background variables was found to have any significant effect. Thus, the variables of pre‐existing chronic disease and multiple viral infections were dropped in a stepwise fashion so that in the final model we only included the variables of age, sex, and treatment intensity. Adjusted odds ratios (aOR) with 95% confidence intervals (CI) were determined. All statistical analyses were carried out using IBM SPSS Statistics for Windows, version 27 (IBM Corp., Armonk, NY).

## 3 RESULTS

### 3.1 Study population characteristics

The median age of 79 study patients at the time of diagnosis was 5 years (interquartile range [IQR] 2, 10; range 0–16 years). Distribution of gender was comparable between treatment intensity groups; overall 40 (51%) patients were girls and 39 (49%) were boys (Table 1).

**TABLE 1:** Patient characteristics across groups.

	*n*/*N* (%)	Low‐intensity treatment (group 1) *n*/*N* (%)	High‐intensity treatment (group 2) *n*/*N* (%)	*p* values
Age at diagnosis in years, median (IQR)	79	4.0 (2.0, 8.0)	7.0 (4.0, 12.0)	.032^a^
Sex, male n/N (%)	39/79 (49%)	26/48 (54%)	13/31 (42%)	.28^b^
Solid tumor	44/79 (56%)	24/48 (50%)	20/31 (65%)	.21^b^
Radiation therapy	13/79 (16%)	0/48	13/31 (42%)	NA
Pre‐existing chronic disease	10/79 (13%)	4/48 (8%)	6/31 (19%)	.18^c^
Multiple respiratory infections previous to diagnosis	7/79 (9%)	2/48 (4%)	5/31 (16%)	.11^c^
Serious infections previous to diagnosis	1/79 (1%)	0/48	1/31 (3%)	NA

*Note*: Main study cohort. Does not include leukemia patients from 2009 to 2013. Chronic disease does not include skin atopic predisposition or allergies.

Abbreviation: IQR, interquartile range.

^a^ Mann–Whitney *U* test.

^b^ Chi‐square.

^c^ Fisher's exact test.

In the high‐intensity treatment group, 42% of patients underwent radiation therapy in addition to cytostatic treatment, while in the low‐intensity treatment group radiation therapy was not delivered. The stratification of ITR‐3^18^ explains this finding, as addition of treatment modalities increases the risk grouping by definition. There were no statistically significant differences relating to the occurrence of multiple respiratory infections or serious infections prior to the diagnosis of malignancy between the groups (Table 1). Diagnoses, applied protocols and patient features are shown in Table S1.

### 3.2 Normalization of immunological parameters

In our dataset including all patients, lymphopenia was more common in the high‐intensity treatment group, as 25/31 (80%) of patients were lymphopenic 1‐month post‐treatment. In the low‐intensity treatment group 15/47 (32%) of patients had lymphopenia at the same time point (*p* < .001). CD4 levels were still low at 4 months post‐treatment in 13/31 (42%) of patients in the high‐intensity treatment group compared to 5/48 (10%) of patients in the low‐intensity treatment group (*p* = .002). NK counts were still low at 4 months post‐treatment for 6/29 (21%) of patients in the high‐intensity treatment group versus 2/45 (4%) of patients in the low‐intensity treatment group (*p* = .05). Patients in the high‐intensity treatment group had significantly lower counts in all immunological parameters compared to the low‐intensity treatment group with the exception of CD19 counts and IgG levels, where no statistically significant differences could be observed (Table 2).

**TABLE 2:** Treatment intensity and lymphocyte subset by 4 months post treatment.

	*n*/*N* (%)	Low‐intensity treatment (group 1) *n*/*N* (%)	High‐intensity treatment (group 2) *n*/*N* (%)	*p* values
Lymphopenia at completion of treatment	40/79 (51%)	15/47 (32%)	25/31 (80%)	<.001^a^
Low CD4	18/79 (23%)	5/48 (10%)	13/31 (42%)	.002^b^
Low NK	8/74 (11%)	2/45 (4%)	6/29 (21%)	.50^b^
Low CD19^c^	3/69 (4%)	2/42 (5%)	1/27 (4%)	1.0^b^
Low IgG^c^	10/71 (14%)	4/44 (9%)	6/27 (22%)	.16^b^

*Note*: Main study cohort. Does not include leukemia patients from 2009 to 2013.

Abbreviations: Low CD19, CD19 count below 90 E6/l by 4 months post‐treatment; Low CD4, CD4 count below 400 E6/l by 4 months post‐treatment; Low IgG, IgG below reference range 4 months post‐treatment; Low NK, NK count below 90 E6/l by 4 months post‐treatment; Lymphopenia, Lymphopenia at completion of treatment (blood samples taken around 1‐month post‐treatment).

^a^ Chi‐square.

^b^ Fisher's exact test.

^c^ Patients having received Rituximab excluded.

In our separate dataset including only patients with leukemia, all of the patients with acute myeloid leukemia (AML) (*N* = 8) showed lymphopenia at 1‐month post‐treatment. In the HR ALL group 4/7 (57%) of patients were lymphopenic and in the SR/ IR ALL group only 2/24 (8%) had lymphopenia at the same time point (*p <* .001). Comparisons between CD4‐, NK‐ and CD19‐ counts and IgG levels between leukemia groups are shown in Table 3. Neutrophil counts had spontaneously normalized by 1‐month post‐treatment in all but 1 patient. This patient was treated according to the HR‐ALL protocol.

**TABLE 3:** Recovery of immunological parameters for leukemia patients.

	Diagnosis			
	ALL SR/IR *n*/*N* (%)	ALL HR *n*/*N* (%)	AML *n*/*N* (%)	*p* values
Low CD4	3/24 (13%)	2/7 (29%)	4/8 (50%)	.080
Low NK	0/23	2/5 (40%)	0/7	NA
Low CD19	2/23 (9%)	1/5 (20%)	0/8	.46^a^
Low IgG	2/24 (8%)	6/7 (86%)	1/8 (13%)	<.001
Lymphopenia	2/24 (8%)	4/7 (57%)	8/8 (100%)	<.001

*Note*: Only leukemia patients analyzed in this data (leukemia study cohort).

Abbreviations: Low CD19, CD19 count below 90 E6/l by 4 months post‐treatment; Low CD4: CD4 count below 400 E6/l by 4 months post‐treatment; Low IgG, IgG below reference range 4 months post‐treatment; Low NK, NK count below 90 E6/l by 4 months post‐treatment; Lymphopenia, Lymphopenia at completion of treatment (blood samples taken at around 1‐month post treatment); P, Fisher's exact test; NA, not applicable.

^a^ *p* value without AML patients.

Adjusted binary logistic regression analysis with background variables (age, sex, treatment intensity, pre‐existing chronic disease, and previous multiple viral respiratory infections) showed a significant effect only for the variable of treatment intensity (Table 4).

**TABLE 4:** Adjusted logistic regression: risk of prolonged time to CD4 count recovery.

Variable	aOR	95% CI	*p* values
Sex	2.83	0.83–9.70	0.10
Age group (pre‐school vs. school)	2.04	0.62–6.72	0.24
Treatment intensity group	6.64	1.88–23.45	0.003

*Note*: Main study cohort.

When analyzing cases stratified by diagnoses, treatment for HR ALL, AML, medulloblastoma, and mature B‐cell lymphoma was associated with prolonged recovery times for a substantial proportion of cases (Tables 3, 5). Some diagnostic groups were represented by only single patients. There was only one patient with ependymoma and one with synovial sarcoma, both with prolonged CD4 recovery (Tables 5 and S1).

**TABLE 5:** Reconstitution of immunological parameters by diagnoses.

ICCC‐3 group	Diagnosis	*N* (%)	Low CD4	Low NK	Low IgG	Low CD19	Lymphopenia
Lymphomas	ALCL	3	0	1 (33%)	0	0	2 (67%)
	Hodgkin lymphoma	5	1 (20%)	0	2 (40%)	0	4 (80%)
	Lymphoblastic lymphoma	2	0	0	0	0	1 (50%)
	Mature B‐cell lymphoma	9	3 (33%)	3 (33%)	4 (44%)	2 (22%)	7 (88%)
CNS tumors	Ependymoma	1	1 (100%)	0	0	0	1 (100%)
	High‐grade glioma	1	0	0	0	0	1 (100%)
	Low‐grade glioma	2	0	0	0	0	0
	Medulloblastoma	5	5 (100%)	2 (40%)	2 (40%)	0	5 (100%)
Neuroblastoma and other peripheral nervous cell tumors	Neuroblastoma	2	0	1 (50%)	0	0	1 (50%)
	Malignant peripheral nerve sheath tumor	1	0	0	0	0	1 (100%)
Retinoblastoma	Retinoblastoma	2	0	0	0	0	0
Renal tumors	Wilms tumor	5	0	0	1 (20%)	0	1 (20%)
Hepatic tumors	Hepatoblastoma	1	0	0	0	0	0
Soft tissue and other extraosseous sarcomas	Rhabdomyosarcoma	3	0	0	0	0	1 (33%)
	Non‐rhabdomyosarcoma	1	1 (100%)	0	0	0	1 (100%)
Germ cell tumors	Germ cell tumor	1	0	0	0	0	0

## 4 DISCUSSION

In our study, most children had normal IgG levels and normal CD19, CD4, and NK counts by 4 months post cancer treatment. Already 1‐month post‐treatment lymphopenia was rare. As expected, there was a marked difference between the low‐intensity treatment group and the high‐intensity treatment group regarding recovery of these parameters. When stratifying according to treatments for specific diagnoses, HR ALL, AML, medulloblastoma, and mature B‐cell lymphoma were associated with prolonged recovery times for a substantial proportion of patients. Within our leukemia cohort, there was a clear contrast between patients with SR/IR ALL and patients with HR ALL or AML regarding their immune recovery profile.

In our cohort, post‐treatment there were no major infectious complications leading to significant morbidity, intensive‐care treatment, or death. However, generally patients were advised to avoid crowded places until their CD4 cell counts had reached a level of 400 E6/l and immunoglobulin production was considered sufficient or immunoglobulin was substituted. Patients with low parameters were also treated using trimethoprim‐sulfamethoxazole as *Pneumocystis jirovecii* prophylaxis. During the first‐year post‐treatment all patients were asked to contact our center in case of a fever. For patients under isolation directions, intravenous antibiotics were always initiated until the etiology of the fever had been more closely evaluated. This study focused on the normalization of immunological parameters rather than infectious symptoms, in order to detect more subtle differences of immune recovery post‐treatment without placing patients at risk.

Several studies have been performed with regard to immune system recovery after treatment for ALL. They suggest profound effects of ALL chemotherapy on the B‐cell compartment, with lesser effects on T‐cells.^5^, ^10^, ^15^, ^17^ There are conflicting results regarding B‐cell recovery. There is evidence of a fast recovery of B‐cell levels^2^, ^5^, ^13^ and a more prolonged process of B‐cell reconstitution on the other.^10^, ^15^, ^17^ B‐cell function, as measured by levels of immunoglobulins, has been shown to recover relatively fast.^2^, ^10^ In comparison to the B‐cell lineage, some studies propose an even more protracted process of recovery for the T‐cell compartment.^11^ NK cells are known to be amongst the first cell populations to recover after stem cell transplantation,^19^ but after non‐transplant treatment for leukemia, van Tilburg et al. showed persisting lower NK cell levels at even 12 months post‐treatment in comparison with healthy controls.^15^ Another study showed fast recovery of NK cell counts, but impaired NK function for several months post‐treatment.^3^ According to the previous literature, it appears that memory B‐ and T‐cells are less affected in comparison to their naïve counterparts,^4^, ^8^, ^16^, ^17^ which explains a relative protection against some earlier‐encountered communicable illnesses.

In comparison to earlier studies, our results from patients with SR and IR ALL suggest a very fast recovery of IgG levels and B‐cell, T‐cell and NK cell numbers. Fast recovery of immunological parameters for these patients in our study may be partly explained by a difference in treatment protocols. In modern protocols, there has been an effort to avoid unnecessary toxicity and to therefore reduce treatment intensity where possible. As an example, prophylactic CNS radiotherapy is not nowadays used in first‐line leukemia treatment. Even so, Kosmidis et al. could not detect any effects of CNS radiotherapy on immune recovery after ALL therapy.^11^ Few previous studies have included patients with non‐transplant HR ALL. The disparities between results for SR/ IR and HR ALL in our study reflect differences in treatment intensity and as such are in line with the previous literature.^4^

Few studies have included non‐hematological malignancies in their assessment of immune reconstitution after cancer therapy.^1^, ^3^, ^6^, ^9^, ^12^, ^14^ Kovacs et al. detected more features of an immune impairment at one‐year post‐treatment in children having been treated for leukemia than in children having undergone therapy for a solid tumor.^12^ Parameters of cellular immunity, antibody‐dependent cellular cytotoxicity, serum immunoglobulin levels and natural killer activity recovered faster in patients with solid tumors, compared to patients having been treated for ALL, while there were no significant differences regarding B‐ and T‐cell blastic transformation.^6^, ^12^ Alanko et al. found the number and function of NK cells to be less affected by cancer therapy for solid tumors than by treatment for ALL.^3^ Hofmann et al. demonstrated differing results for their cohort of patients with ALL, Hodgkin lymphoma and Ewing sarcoma.^9^ Since treatments for solid tumors are very heterogenous, we did not categorize between diagnoses of leukemia or solid tumors per se, but rather divided patients into those with low‐intensity treatment or high‐intensity treatment. We used the ITR‐3 as a stratifying tool. It must be noted that the treatment modality of radiotherapy raises the ITR‐3 classification in our study to high‐intensity treatment.^18^ There are previous reports to support a long‐lasting immunosuppressive effect of radiotherapy,^1^, ^12^ and particularly prolongation of T‐cell recovery in radiotherapy‐treated patients.^1^ However, it is clear that the challenge posed by heterogeneity of treatments could not be entirely solved by the application of ITR‐3 treatment intensity grading.

Young children constitute a vulnerable population with regard to infections, since acquired immunity is still developing. According to our findings, the age of the patient did not affect immune reconstitution. Some earlier studies found patients of young age to display prolonged immune disturbances^14^ or they indicated varying results with respect to different immunological parameters.^11^ Others showed no effect of patient age.^16^ Thymus‐dependent production of T‐lymphocytes is an age‐dependent process particularly implicated for the recovery of CD4 lymphocytes, which has greatest regenerative potential during early childhood and declines with older age.^20^, ^21^ However, thymus‐dependent processes have been shown to continue into adult age.^20^

We consider the main strength of this study is the real‐life follow‐up of a large variety of pediatric malignancy patients stratified according to treatment intensity as per ITR‐3. Previous studies have often evaluated small‐size homogenous treatment groups, which allows in‐depths immunological analyses, but fails to elicit trends between different diagnostic and treatment subsets. When considering potential confounders, it must be borne in mind that the vast majority of our patients were of Scandinavian origin, which means little population‐based genetic variation of immune mechanisms, and minimal cultural and language diversity relating to practical aspects of patient care.

The main limitation of this study consists in the fact that – due to the retrospective nature of this work—functional analyses could not be carried out, and a more detailed immunological assessment was precluded. It has to be borne in mind that some degree of functional impairment may be present for a prolonged time, even after quantitative cell parameters have returned to the normal range. We did not have the possibility to perform a detailed chronological or long‐term follow‐up of our patients. When analyzing the high‐intensity treatment group, we found patients to show lymphopenia 1‐month post‐ treatment in 80% of cases. By 4 months post‐treatment, CD4 lymphocyte counts were low in only 42% of cases. There was a similar, but even steeper decline of those numbers for the low‐intensity treatment group. Although lymphopenia cannot be directly assumed for all patients with low CD4 counts and vice versa, it appears that those cell count numbers normalized within the time frame of 1–4 months for a considerable proportion of patients.

Our results show a rapid recovery of immunological parameters for most cases and thereby suggest that isolation precautions are unnecessary after cessation of treatment in many patients. Within the high‐intensity treatment group prolonged recovery of immunological parameters was seen especially for HR ALL, AML, medulloblastoma, and mature B‐cell lymphoma. These patients need special attention and follow‐up after completion of childhood cancer treatment.

## AUTHOR CONTRIBUTIONS

Ella Antikainen: investigation, methodology, formal analysis, writing original draft. Marika Grönroos: conceptualization, methodology, writing – review and editing. Anu Huurre: conceptualization, methodology, writing – review and editing. Laura Korhonen: conceptualization, methodology, writing – review and editing. Ville Peltola: conceptualization, methodology, writing – review and editing, supervision. Päivi Lähteenmäki: conceptualization, methodology, writing – review and editing, resources, project administration, supervision. Schuez‐Havupalo Linnea: conceptualization, methodology, formal analysis, writing of original draft and review/editing, project administration, supervision, funding acquisition.

## CONFLICT OF INTEREST STATEMENT

The authors have stated explicitly that there are no conflicts of interest in connection with this article.

## ETHICS STATEMENT

All the data analyzed were collected as part of routine diagnostics and treatment. Data analysis was carried out in connection with a quality evaluation procedure and did therefore not require ethical approval (permission to use hospital data was granted from the hospital review board, permit number TO8/027/19). Data were handled in a strictly anonymous manner.

## Supporting information


**TABLE S1.** ICCC‐3 groups, diseases, applied protocols, features of patients with prolonged recovery of CD4 lymphocyte subsets.

## Data Availability

The data that support the findings of this study are available on request from the corresponding author. The data are not publicly available due to privacy or ethical restrictions.

## References

[1] Alanko S , Pelliniemi T‐T , Salmi TT . Recovery of blood lymphocytes and serum immunoglobulins after treatment of solid tumors in children. Pediatr Hematol Oncol. 1994;11(1):33‐45. doi:10.3109/08880019409141899 8155498

[2] Alanko S , Pelliniemi T‐T , Salmi TT . Recovery of blood B‐lymphocytes and serum immunoglobulins after chemotherapy for childhood acute lymphoblastic leukemia. Cancer. 1992;69(6):1481‐1486. doi:10.1002/1097-0142(19920315)69:6<1481::aid-cncr2820690628>3.0.co;2-l 1540885

[3] Alanko S , Salmi TT , Pelliniemi T‐T . Recovery of natural killer cells after chemotherapy for childhood acute lymphoblastic leukemia and solid tumors. Med Pediatr Oncol. 1995;24(6):373‐378. doi:10.1002/mpo.2950240607 7715543

[4] Ek T , Mellander L , Andersson B , Abrahamsson J . Immune reconstitution after childhood acute lymphoblastic leukemia is most severely affected in the high risk group. Pediatr Blood Cancer. 2005;44(5):461‐468. doi:10.1002/pbc.20255 15558707

[5] Eyrich M , Wiegering V , Lim A , Schrauder A , Winkler B , Schlegel PG . Immune function in children under chemotherapy for standard risk acute lymphoblastic Leukaemia—a prospective study of 20 paediatric patients. Br J Haematol. 2009;147(3):360‐370. doi:10.1111/j.1365-2141.2009.07862.x 19694715

[6] Gadó J , Schlick B , Bárány O , et al. Az immunrendszer allapota gyermekkori malignus daganatok terápiáját követoen (the function of the immune system after the treatment of pediatric malignant diseases). Orv Hetil. 2006;147(36):1731‐1738. 17087017

[7] Guilcher GMT , Rivard L , Huang JT , et al. Immune function in childhood cancer survivors: a Children's oncology group review. Lancet Child Adolesc Health. 2021;5(4):284‐294. doi:10.1016/S2352-4642(20)30312-6 33600774 PMC8725381

[8] Haining WN , Neuberg DS , Keczkemethy HL , et al. Antigen‐specific T‐cell memory is preserved in children treated for acute lymphoblastic leukemia. Blood. 2005;106(5):1749‐1754. doi:10.1182/blood-2005-03-1082 15920008 PMC1895221

[9] Hofmann G , Zierk J , Sobik B , et al. Temporal evolution and differential patterns of cellular reconstitution after therapy for childhood cancers. Sci Rep. 2023;13(1):4022. doi:10.1038/s41598-023-31217-3 36899075 PMC10006072

[10] Koskenvuo M , Ekman I , Saha E , et al. Immunological reconstitution in children after completing conventional chemotherapy of acute lymphoblastic leukemia is marked by impaired B‐cell compartment. Pediatr Blood Cancer. 2016;63(9):1653‐1656. doi:10.1002/pbc.26047 27163649

[11] Kosmidis S , Baka M , Bouhoutsou D , et al. Longitudinal assessment of immunological status and rate of immune recovery following treatment in children with all. Pediatr Blood Cancer. 2008;50(3):528‐532. doi:10.1002/pbc.21327 17853465

[12] Kovacs GT , Barany O , Schlick B , et al. Late immune recovery in children treated for malignant diseases. Pathol Oncol Res. 2008;14(4):391‐397. doi:10.1007/s12253-008-9073-5 18575827

[13] Mazur B , Szczepański T , Karpe J , Sońta‐Jakimczyk D , Bubała H , Torbus M . Decreased numbers of CD4+ T lymphocytes in peripheral blood after treatment of childhood acute lymphoblastic leukemia. Leuk Res. 2006;30(1):33‐36. doi:10.1016/j.leukres.2005.05.024 16039713

[14] Mustafa MM , Buchanan GR , Winick NJ , et al. Immune recovery in children with malignancy after cessation of chemotherapy. J Pediatr Hematol Oncol. 1998;20(5):451‐457. doi:10.1097/00043426-199809000-00008 9787318

[15] van Tilburg CM , van der Velden VHJ , Sanders EAM , et al. Reduced versus intensive chemotherapy for childhood acute lymphoblastic leukemia: impact on lymphocyte compartment composition. Leuk Res. 2011;35(4):484‐491. doi:10.1016/j.leukres.2010.10.005 21051085

[16] van Tilburg CM , van Gent R , Bierings MB , et al. Immune reconstitution in children following chemotherapy for haematological malignancies: a long‐term follow‐up. Br J Haematol. 2010;152(2):201‐210. doi:10.1111/j.1365-2141.2010.08478.x 21114483

[17] Wiegering V , Frank J , Freudenberg S , et al. Impaired B‐cell reconstitution in children after chemotherapy for standard or medium risk acute precursor B‐lymphoblastic leukemia. Leuk Lymphoma. 2013;55(4):870‐875. doi:10.3109/10428194.2013.816423 23786458

[18] Kazak AE , Hocking MC , Ittenbach RF , et al. A revision of the intensity of treatment rating scale: classifying the intensity of pediatric cancer treatment. Pediatr Blood Cancer. 2012;59(1):96‐99. doi:10.1002/pbc.23320 21858914 PMC3223269

[19] Kalwak K , Gorczyńska E , Toporski J , et al. Immune reconstitution after haematopoietic cell transplantation in children: immunophenotype analysis with regard to factors affecting the speed of recovery. Br J Haematol. 2002;118(1):74‐89. doi:10.1046/j.1365-2141.2002.03560.x 12100130

[20] Mackall CL , Fleisher TA , Brown MR , et al. Age, thymopoiesis, and CD4+ T‐lymphocyte regeneration after intensive chemotherapy. N Engl J Med. 1995;332(3):143‐149. doi:10.1056/nejm199501193320303 7800006

[21] Mackall CL , Fleisher TA , Brown MR , et al. Distinctions between CD8+ and CD4+ T‐cell regenerative pathways result in prolonged T‐cell subset imbalance after intensive chemotherapy. Blood. 1997;89(10):3700‐3707. doi:10.1182/blood.v89.10.3700.3700_3700_3707 9160675

[22] Jakacki RI , Cohen KJ , Buxton A , et al. Phase 2 study of concurrent radiotherapy and temozolomide followed by temozolomide and lomustine in the treatment of children with high‐grade glioma: a report of the Children's oncology group ACNS0423 study. Neuro‐Oncology. 2016;18(10):1442‐1450. 27006176 10.1093/neuonc/now038 PMC5035517

