# Supplementary material for: Treatment intensity affects immune reconstitution even after childhood cancer not treated with hematopoietic stem cell transplantation
Source: Cancer Rep (Hoboken). 2024 May 20;7(5):e2069. doi: 10.1002/cnr2.2069 (PMC11104287; doi:10.1002/cnr2.2069)
Supplement: Supplementary file 1 — TABLE S1. ICCC‐3 groups, diseases, applied protocols, features of patients with prolonged recovery of CD4 lymphocyte subsets. [file CNR2-7-e2069-s001.docx]

| **TABLE S1**  **ICCC-3 groups, diseases, applied protocols, features of patients with prolonged recovery of CD4 lymphocyte subsets.** | | | | |
| --- | --- | --- | --- | --- |
| ICCC-3 group | Disease | Applied protocols and details on radiotherapy | Patients with prolonged CD4 recovery (n/N) | Additional features of patients with prolonged CD4 recovery |
| Leukemias | ALL | Interfant 06 | 0/1 |  |
|  |  | NOPHO ALL 2008 | 5/29 | 3 IR patients aged 13, 15 and 16 (of which 1 Down sdr patient); 2 HR patients aged 14 and 15. |
|  | Philadelphia ALL | EsPhALL2010/ NOPHO version | 0/1 |  |
|  | AML | NOPHO-DBH-AML2012 | 4/7 |  |
|  |  | Down AML 2007 | 0/1 |  |
| Lymphomas | Anaplastic large cell lymphoma | ALCL-99  B-NHL 2013 (BFM and NOPHO) | 0/2  0/1 |  |
|  | Hodgkin’s lymphoma | EuroNet-PHL-C1 2006 (2 patients with radiotherapy 30-31gy) | 1/5 | TG2 and radiotherapy 31gy |
|  | Mature B-cell lymphoma | B-NHL 2013 (BFM and NOPHO) | 1/7 | R2 |
|  |  | BFM-NHL 2004 | 1/1 | R4 |
|  |  | FAB/LMB96 | 1/1 | Group B |
|  | Lymphoblastic lymphoma | Euro-LBL2014 | 0/2 |  |
| CNS tumors | Low grade glioma | LGG 2004 | 0/2 |  |
|  | High grade glioma | Radiotherapy (60gy); temozolamide/ lomustine b)  subsequently PCV-treatment. c) | 0/1 |  |
|  | Medulloblastoma | SIOP-PNET5MB | 5/5 | CSI |
|  | Ependymoma | SIOP ependymoma | 1/1 | CSI |
| Neuroblastoma and other peripheral nervous cell tumors | Neuroblastoma | LINES protocol SIOPEN 2010 | 0/2 |  |
|  | Malignant peripheral nerve sheath tumor | CWS guidance 2014 (radiotherapy 50gy) | 0/1 |  |
| Retinoblastoma | Retinoblastoma | VEC-treatment a) | 0/2 |  |
| Renal tumors | Wilms tumor | Umbrella protocol SIOP-RTSG 2016 (1 patient with radiotherapy 26gy) | 0/5 |  |
| Hepatic tumors | Hepatoblastoma | SIOPEL-6 2008 | 0/1 |  |
| **Soft tissue and other extraosseous sarcomas** | Rhabdomyosarcoma | CWS guidance 2014 (1 patient with protone therapy to orbit) | 0/2 |  |
|  |  | CWS guidance 2006 | 0/1 |  |
|  | Synovial sarcoma | CWS guidance 2014 | 1/1 | IRS2A; radiotherapy 31 gy |
| **Germ cell tumors** | Germ cell tumor | GCT-III 2005 | 0/1 |  |
| **Note. All study patients included.**   1. VEC treatment: vincristine, etoposide, carboplatin 2. Jakacki R et al.^22^ 3. procarbazine, lomustine, vincristine | | | | |
